# Repair Rate and Associated Costs of Reusable Flexible Ureteroscopes: A Systematic Review and Meta-analysis

**DOI:** 10.1016/j.euros.2021.12.013

**Published:** 2022-01-29

**Authors:** Dinah K. Rindorf, Thomas Tailly, Guido M. Kamphuis, Sara Larsen, Bhaskar K. Somani, Olivier Traxer, Kevin Koo

**Affiliations:** a Ambu A/S, Ballerup, Denmark; b Department of Urology, University Hospital Ghent, Ghent, Belgium; c Department of Urology, Amsterdam UMC, Amsterdam, The Netherlands; d Department of Urology, University Hospital Southampton NHS FT, Southampton, UK; e Department of Urology, Tenon Hospital, Sorbonne University, Paris, France; f Department of Urology, Mayo Clinic, Rochester, MN, USA

**Keywords:** Equipment failure, Health care costs, Ureteroscopes, Ureteroscopy

## Abstract

**Context:**

The refined mechanics of a flexible ureteroscope (fURS) are vulnerable to damage. Sending the fURS for repair is costly and has driven interest toward estimating the resources used for fURS repairs.

**Objective:**

To systematically review available literature and to estimate the total weighted repair rate of an fURS and the average repair cost per ureteroscopy.

**Evidence acquisition:**

A systematic review was conducted by searching the MEDLINE, Embase, Web of Science, and Cochrane Library databases. The average costs of all repairs identified in the included studies were extracted. A random-effect model was used to calculate the pooled total fURS repair rate. The total weighted repair rate and average cost per repair were multiplied to provide an average cost of repair per ureteroscopy procedure.

**Evidence synthesis:**

We identified 18 studies that fulfilled the inclusion criteria, which included 411 repairs from 5900 investigated ureteroscopy procedures. The calculated weighted repair rate was 6.5% ± 0.745% (95% confidence interval: 5.0–7.9%; I^2^ = 75.3%), equivalent to 15 ureteroscopy procedures before repair. The average cost per repair was 6808 USD; according to the weighted repair rate of 6.5%, this corresponds to an average repair cost of 441 USD per procedure. Egger’s regression test did not reveal a significant publication bias (*p* = 0.07).

**Conclusions:**

This is the first meta-analysis to estimate the repair rate of the fURS used for ureteroscopy. Our analysis demonstrates a repair rate of 6.5%, equivalent to 15 ureteroscopy procedures between fURS repairs and a repair cost of 441 USD per procedure. Ureteroscopy practices should consider fURS breakage rates and repair costs to optimize the use of reusable versus disposable devices.

**Patient summary:**

We reviewed available literature investigating the repair rate of a flexible ureteroscope (fURS). We found that fURSs are sent for repair after every 15 ureteroscopy procedures, corresponding to 441 USD per procedure in repair cost.

## Take Home Message

Our analysis demonstrates a repair rate of 6.5%, equivalent to 15 ureteroscopy procedures between flexible ureteroscope repairs and a repair cost of 441 USD per procedure.

## 1 Introduction

Ureteroscopy is the most commonly performed procedure for nephrolithiasis worldwide [1] due to its broad, guideline-recommended indications [2], [3]. Besides the use for nephrolithiasis, ureteroscopy is used for the screening and treatment of urothelial cancer, as well as for the treatment of stricture disease. Ureteroscopes are vulnerable to damage sustained during procedures or reprocessing after use. Frequent reusable flexible ureteroscope (fURS) damage necessitates routine repair, which is costly and makes the device unavailable for clinical use. Decontamination and sterilization require specialized equipment and well-trained staff to avoid straining the device’s mechanics, and inadequate decontamination increases the risk of infection transmission [4], [5], [6].

Single-use fURSs have been introduced to overcome these disadvantages. Commercially available single-use fURSs have comparable and, in some cases, superior performance to reusable fURSs [7], [8], [9], [10], [11]. Considerations on the cost effectiveness of these single-use alternatives arise given limited resources in the health care sector. Several single-institution studies have compared the costs of reusable and single-use fURSs [12], [13], [14], [15], [16], [17], [18], [19], [20]. The majority of these studies highlighted the significant expenditures associated with frequent repairs that, in some studies, account for half of the total costs of maintaining a fleet of fURSs necessary for clinical practice [12], [19].

With the introduction of single-use fURSs, it is especially relevant to perform an accurate assessment of the resources required for the maintenance and repair of an fURS. While studies have investigated and documented the durability of reusable fURSs, none has determined overall repair rates and associated per-procedure costs based on all published studies. The aim of this work was to systematically review available literature and estimate (1) the total weighted repair rate of reusable fURSs and (2) the average repair cost per ureteroscopy.

## 2 Evidence acquisition

A systematic review of published literature on repair rates for fURSs was conducted according to Preferred Reporting Items for Systematic Reviews and Meta-analyses (PRISMA) guidelines. A protocol was registered in PROSPERO (no. CRD42020207307). The systematic search was carried out in the PubMed, Embase, and Web of Science databases and the CENTRAL trial registry of the Cochrane Collaboration. The search was limited to English-language publications involving humans. To ensure literature saturation, reference lists of included studies were screened for eligibility. The search was performed on April 2, 2021.

The full search string in PubMed was (((ureteroscop* OR (retrograde AND intrarenal AND surger*) OR rirs OR furs OR ureterorenoscop*) AND (repair* OR cost*)) NOT (animals[MeSH Terms] NOT humans[MeSH Terms])) AND (English [language]). The search string was adapted to fit the search parameters of each database.

### 2.1 Inclusion and exclusion criteria

A search was conducted to identify all relevant studies assessing the fURS repair rate associated with ureteroscopies (primary outcome). For inclusion, studies needed to state the total number of reusable fURS repairs needed out of a total number of identified ureteroscopy procedures where an fURS was used. This information was collected to estimate the repair rate for reusable fURSs. Repair was defined as a reusable fURS in need of repair due to a defect following one or several ureteroscopies. Therefore, studies addressing “defects” (or similar wording) instead of “repairs” were excluded if the study failed to account for whether or not the defect involved a repair order. Study inclusion was not limited by the publication year. The secondary outcome for this systematic search was repair cost per order reported as the average cost per order of all the repairs ordered during the study period.

Studies with fewer than 50 samples were excluded. A minimum sample of 50 procedures was selected after an initial search showed an average number of 20 procedures before repair, so 50 procedures was selected to ensure a minimum observation of two repairs within the study period. Studies conducted in academic centers where the use of the fURS was not limited to trainees were eligible for review. Further exclusion criteria were animal studies, conference abstracts or proceedings, case reports, editorials, commentaries, and systematic or narrative reviews. However, systematic reviews were retained for discussion and as a source of potentially relevant studies in the reference list.

### 2.2 Data extraction

Studies found in this search were uploaded to the reference tool Mendeley Desktop, version 1.19.4 (Mendeley Ltd, London, UK). Duplicates were removed. Identified titles and abstracts were reviewed, and relevant articles were selected for full-text review if they met all the inclusion criteria. The titles and abstracts of the identified studies were independently reviewed by two authors (D.R. and S.L.). Studies that did not fulfill the abovementioned criteria were excluded, and disagreements were resolved by consensus or by consultation with a third author (K.K.).

### 2.3 Outcomes

The primary study outcome of the pooled analysis was the total weighted repair rate based on the number of repairs among all sampled ureteroscopies. Secondary outcomes were costs associated with sending the ureteroscopes for repair, reported as the average cost per order of repair. All repair costs were adjusted for inflation to 2020 values according to national Consumer Price Indexes provided by the Organisation for Economic Co-operation and Development (available at https://data.oecd.org/). A subgroup analysis was performed to assess the rate of major repairs, as it was assumed that major repairs differ substantially from minor repairs in terms of costs and potential impacts on hospital activities. Data on major repairs were collected for studies distinguishing between the major and minor repairs. Major repairs were defined as significant damage compromising the function of the fURS requiring repair cost of >5156 USD. Thus, studies stating the number of major repairs compared with that of minor repairs were included for this subgroup analysis. The extracted data variables were author, country, study design, surgical technique, study period, reusable fURS brand, number of ureteroscopy procedures investigated, number of repairs (major and minor), type of fURS (fiberoptic or digital), research setting, and average cost per repair order if applicable (Table 1). Two studies [21], [22] investigated the differences in repair rates between two groups to compare differences in outcomes. For these studies, data were pooled to provide a combined estimate of the number of repairs among all sampled ureteroscopies.

**Table 1:** Baseline characteristics of the included studies

	Author (year)	Country	Study design	Study period	Brand	No. of procedures	No. of repairs	Repair cost ($) a	No. of major/minor repair	Cost of major/minor repair ($) a	Fiber or digital	Sheath use^b^	Setting
1.	Van Compernolle et al (2021) [32]	BE	R	2016–2020	Karl Storz	983	45	6781	22/9	12 307/3221	D	–	Academic hospital
2.	Banerjee et al (2021) [33]	USA	R	2017–2019	Karl Storz	1211	143	9754	137/6	10 132/332	D	–	Academic hospital & ambulatory surgery center
3.	Kam et al (2019) [11]	AUS	P	2016–2017	Olympus	64	4	–	4/0	≈7914/–	D	71.0	Academic hospital
4.	Hennessey et al (2018) [34]	AUS	P	–	Olympus	234	15	7817	15/0	>7330/<7330	D	100.0	Academic hospital
5.	Mager et al (2018) [13]	DE	P	2015–2016	Karl Storz	68	9	–	–	–	F, D	–	Academic hospital
6.	Taguchi et al (2018) [35]	USA	P	2014–2015	Olympus, Karl Storz	424	28	–	–	–	F, D	44.8	Academic hospital
7.	Martin et al (2017) [14]	USA	P	2014–2015	Karl Storz	160	11	–	–	–	D	68.1	Academic hospital
8.	Ozimek et al (2017) [19]	DE	R	2013–2016	Olympus, Karl Storz	432	32	3871	–	–	F, D	25.0	Academic hospital
9.	Kramolowsky et al (2016) [25]	USA	R	2011–2014	Olympus	655	31	8325	–	–	F	13.4	Community hospital (ambulatory surgical center)
10.	Shah et al (2015) [26]	USA	P	–	Olympus	101	8	–	8/0	–	D	86.0	Academic hospital
11.	Tosoian et al (2015) [27]	USA	R	2013	–	190	20	6257	–	–	F	–	Academic hospital
12.	Gurbuz et al (2014) [15]	TR	R	2010–2013	Karl Storz	302	6	6701	4/2	9324/2016	–	–	Academic hospital
13.	Karaolides et al (2013) [21]	UK	P	2011	Olympus	141	8	–	8/0	–	D	68.8	Academic hospital
14.	Khan et al (2013) [22]	UK	P	2010–2011	Gyrus ACMI, Karl Storz	193	10	6638	–	–	F	–	Academic hospital
15.	Somani et al (2011) [28]	UK	P	2009–2010	Karl Storz	260	11	5131	10/1	5156/2234	–	–	Academic hospital
16.	Binbay et al (2010) [29]	TR	P	2008–2009	Gyrus ACMI, Karl Storz	76	4	–	–	–	F, D	39.5	Academic hospital
17.	Carey et al (2006) [30]	USA	P	2001–2004	Gyrus ACMI, Karl Storz	324	17	–	–	–	F	–	Academic hospital
18.	Afane et al (2000) [31]	USA	P	1997–1999	Karl Storz, Circon Gyrus ACMI, Wolf, Olympus	92	9	–	–	–	F	23.8	Academic hospital

AUS = Australia; BE = Belgium; D = digital ureteroscope; DE = Germany; F = fiberoptic ureteroscope; P = prospective; R = retrospective; TR = Turkey.

a Average repair cost per order. Costs are adjusted for inflation to present value of 2020 according to national Consumer Price Indexes according to the Organisation for Economic Co-operation and Development and converted from local currency to USD.

b Sheath use in percentage of total procedures.

### 2.4 Data analysis and statistical methods

We performed a meta-analysis of the repair rate of fURS used for ureteroscopies. In addition to the subgroup analyses of major repairs, subgroup analyses were performed for studies using digital fURSs, US studies, and studies published in the past decade (after the year 2011) as it was assumed that these factors could significantly influence the repair rate. Data were pooled using a random-effect model based on proportions. This approach was selected a priori to adjust for the heterogeneity of patient populations and techniques. Statistical heterogeneity was measured with the random-effect variance (t^2^) and I^2^ statistics [23], which quantify inconsistency across studies. Values below 25% were considered indicative of low heterogeneity [23]. Publication bias is known to impact the validity and generalizability of conclusions based on meta-analyses [24]. Publication biases were assessed using funnel plots, and funnel asymmetry was assessed with Egger’s regression tests. A forest plot of all outcomes was created using the random-effect model. All analyses were done using the standard software package Stata/SE, version 16.1 (StataCorp LLC, College Station, TX, United States).

## 3 Evidence synthesis

### 3.1 Characteristics of included studies

**Fig. 1:**
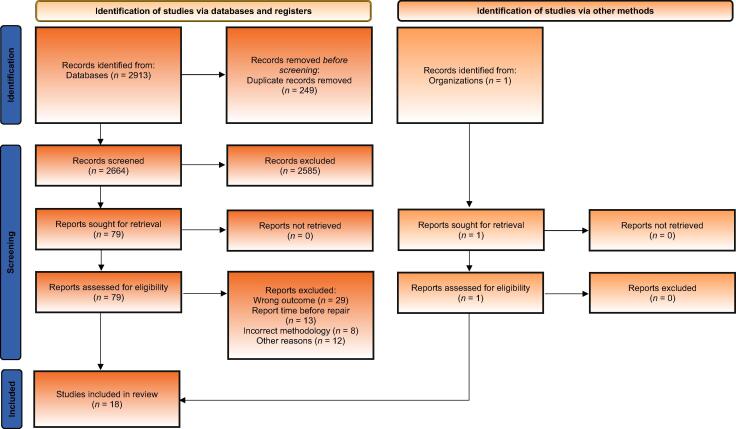
PRISMA flowchart of the study search and publication screening and selection. PRISMA = Preferred Reporting Items for Systematic Reviews and Meta-analyses.

Figure 1 shows the number of articles screened, number of articles accessed for eligibility, and reasons for exclusion. Eighteen studies were selected for the final analysis. All the 18 studies were published between 2000 and 2021, and included ureteroscopy procedures performed in the period between December 1997 and March 2020. The included studies yielded a total sample size of 5900 ureteroscopy procedures, and there were 411 repairs on reusable fURSs used for these procedures.

The baseline characteristics of the included studies are shown in Table 1. Most (*n* = 17, 94.4%) took place in academic hospitals [11], [13], [14], [15], [19], [21], [22], [26], [27], [28], [29], [30], [31], [32], [33], [34], [35]. Twelve studies were prospective [11], [13], [14], [21], [22], [26], [28], [29], [30], [31], [34], [35] and six were retrospective [15], [19], [25], [27], [32], [33]. Six of the studies were from Europe [13], [19], [21], [22], [28], [32], eight were from the USA [14], [25], [26], [27], [30], [31], [33], [35], and four were from other regions (Turkey and Australia) [11], [15], [29], [34]. Twelve studies used Karl Storz reusable fURS [13], [14], [15], [19], [22], [28], [29], [30], [31], [32], [33], [35], eight used Olympus reusable fURS [11], [19], [21], [25], [26], [31], [34], [35], and four used Gyrus ACMI [22], [29], [30], [31]. Seven of the studies used digital reusable fURSs for the procedures [11], [14], [21], [26], [32], [33], [34], five used fiberoptic fURSs [22], [25], [27], [30], [31], four used both fiberoptic and digital fURSs [13], [19], [29], [35], and two did not report the type of reusable fURSs [15], [28]. Finally, eight of the included studies distinguished between major and minor repairs [11], [15], [21], [26], [28], [32], [33], [34], ten reported how often a sheath was used [11], [14], [19], [21], [25], [26], [29], [31], [34], [35], and nine reported the average repair cost per order [15], [19], [22], [25], [27], [28], [32], [33], [34].

### 3.2 Primary outcome analysis

**Fig. 2:**
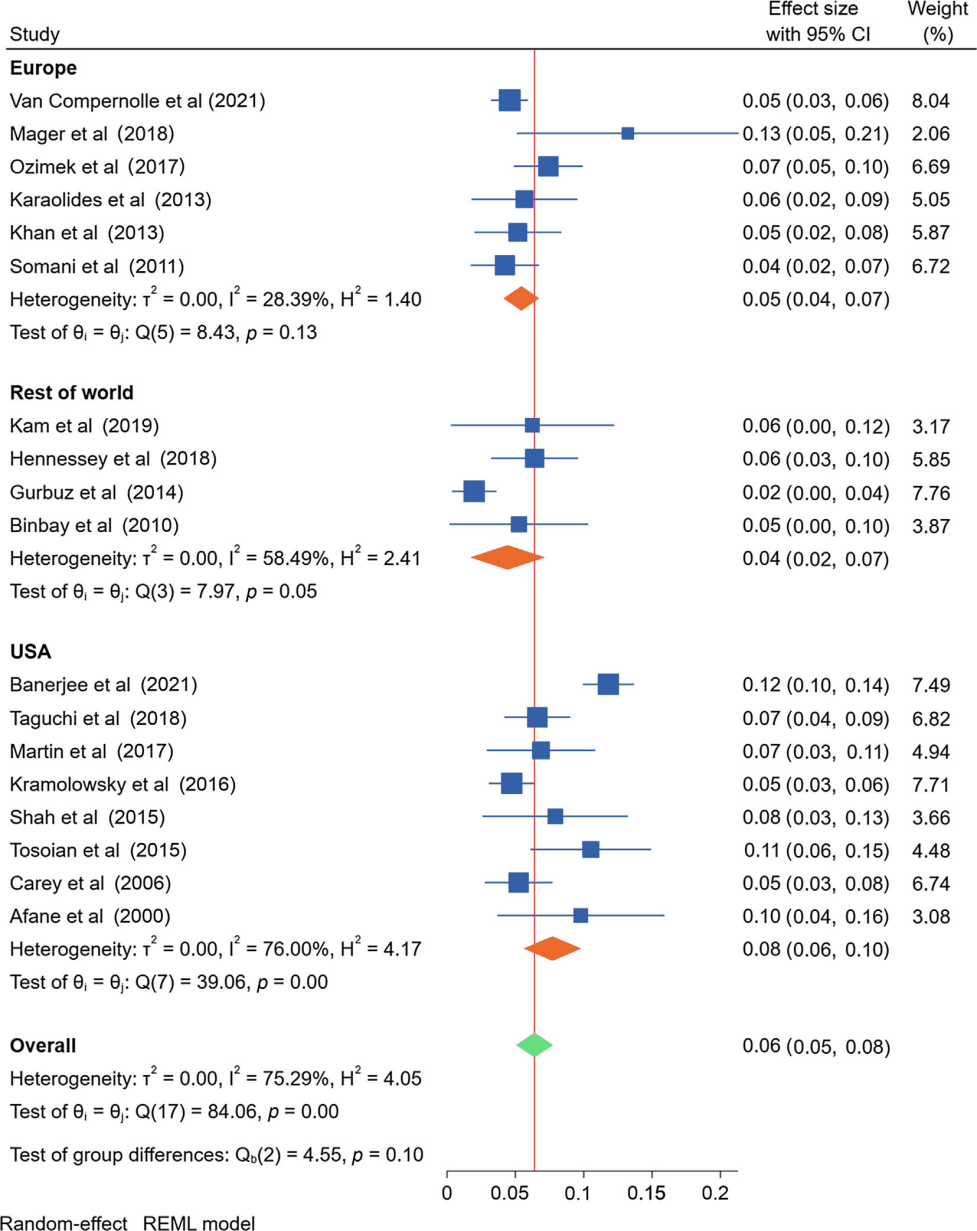
Forest plot of the overall pooled estimate of fURS repair rate and pooled estimates of fURS repair rates by region. The green diamond represents the overall 95% CI. (For interpretation of the references to colour in this figure legend, the reader is referred to the web version of this article.)

**Fig. 3:**
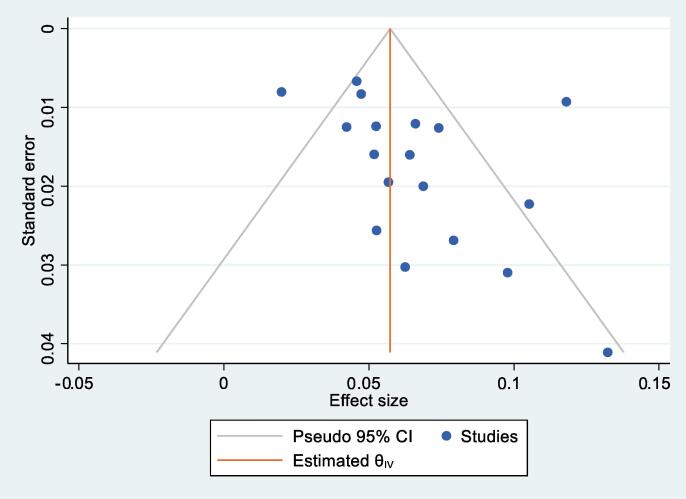
A funnel plot of the overall pooled repair rate of fURS for visual inspection. CI = confidence interval; fURS = flexible ureteroscope.

The meta-analysis of the included studies demonstrated an overall pooled repair rate of 6.5% ± 0.745% (95% confidence interval [CI]: 5.0–7.9%; I^2^ = 75.3%), equivalent to 15 ureteroscopy procedures (95% CI: 13–20) before repair (Fig. 2). Visual inspection of the funnel plot was not suggestive of a publication bias (Fig. 3), and Egger’s regression test of the hypothesis of no small study effects did not indicate a significant publication bias (*p* = 0.07). However, the heterogeneity across the 18 included studies was considered high. The average cost per repair was 6808 USD; according to the weighted repair rate of 6.5%, this corresponds to an average repair cost of 441 USD per procedure.

### 3.3 Subgroup analyses

A meta-analysis of the included studies assessing major repairs demonstrated a pooled major repair rate of 5.5% ± 1.434% (95% CI: 2.6–8.3%; I^2^ = 91.2%). A meta-analysis of studies using digital reusable fURSs revealed a pooled repair rate of 7.2% ± 0.983% (95% CI: 5.3–9.2%; I^2^ = 67.5%). An independent group *t* test showed that the repair rate for a digital fURS was not significantly higher than that of a fiberoptic fURS (*p* = 0.48). Heterogeneity among the studies included in the subgroup analyses for major repairs and for digital versus fiberoptic fURSs was considered high. A meta-analysis of studies from the USA (*n* = 8) demonstrated a pooled repair rate of 7.8% ± 1.221% (95% CI: 5.4–10.2%; I^2^ = 75.8%). Studies published within the past decade (*n* = 14) reported a repair rate of 6.7% ± 0.899% (95% CI: 5.0–8.5%). Heterogeneity was considered high in both subgroup analyses for the US studies and those published within the past decade. Finally, a meta-analysis of studies with sheath use in the majority (>50%) and minority (<50%) of cases revealed a pooled repair rate of 6.5% ± 0.932% (95% CI: 4.7–8.3%; I^2^ = 0.11%) and 6.1% ± 0.730% (95% CI: 4.7–7.6%; I^2^ = 31.5%). For these subgroup analyses, the heterogeneity was considered low to moderate. Forest plots of the pooled estimate of fURS repair rate for subgroups can be found in Supplementary material.

### 3.4 Discussion

This is the first systematic literature review and meta-analysis to estimate the repair rate of reusable fURSs used for ureteroscopy procedures. The results showed a 6.5% repair rate, equivalent to 15 ureteroscopy procedures before repair. The average cost per repair was 6808 USD; according to the weighted repair rate of 6.5%, this corresponds to an average repair cost of 441 USD per procedure.

In health care systems with limited resources, cost is an important issue with implications for value and efficiency. Several studies estimated the per-procedure cost of ureteroscopy with reusable fURSs with the aim of comparing it with the cost of single-use ureteroscopes [12], [13], [14]. Three elements should be considered during this comparison: (1) repair costs of reusable fURSs, (2) initial purchase price of reusable fURSs, and (3) costs associated with the reprocessing and maintenance of reusable fURSs (including labor time and capital investments in equipment).

In addition to high repair costs, there are negative impacts on clinical practice. Damage to reusable endoscope channels secondary to device manipulation during procedures or reprocessing may increase the potential for biofilm formation that has been associated with endoscopy-related infections [5], [36]. Kovaleva et al [36] concluded that damaged endoscopes were one of the most common factors associated with microbiological transmission during gastrointestinal endoscopy. Legemate et al [6] found that 12.1% of their preuse ureteroscope cultures were positive; however, none of the patients who underwent surgery with a uropathogen-contaminated ureteroscope developed urinary tract infections. The U.S. Food and Drug Administration recently announced an investigation of patient infections and other possible contamination issues associated with reprocessing urological endoscopes after receiving 450 medical device reports describing postprocedure patient infections or other possible contamination issues between January 1, 2017 and February 20, 2021. Although the Food and Drug Administration is in the early stages of evaluation, it believes that the risk of infection is low based on the available data [37]. In situations where there is heightened concern about infection due to specific patient or clinical factors, single-use equipment may help mitigate the risks of endoscope-associated infection.

Since fURS repair is costly and is needed after every 15 ureteroscopy procedures according to our findings, close consideration should be paid to techniques and strategies in clinical practice to increase the durability of reusable fURSs. Two groups demonstrated that the reprocessing cycle after use can damage an fURS [38], [39]. Although damage necessitating repair may occur during reprocessing, two studies estimated that it occurs rather infrequently [30], [40], and one study concluded that the technique and number of personnel involved in fURS reprocessing did not have a significant effect on reusable fURS durability [41]. However, Sooriakumaran et al [42] concluded that a staggering 72% of repairs during the 1-yr investigation period were most likely for the damage incurred while reprocessing*.* Owing to the high risk and prevalence of fURS damage during reprocessing, some authors underscored the importance of having dedicated, well-trained staff in charge of this task [30], [40]. However, training programs for surgeons or equipment maintenance staff may require additional resources. It is therefore necessary to balance personnel training needed to maintain a fleet of reusable fURSs and the reprocessing-related risk of fURS damage.

Our study revealed similar repair rates for the procedures performed with a ureteral access sheath (UAS) in the majority versus minority of cases (6.5% vs 6.1%). Other studies have confirmed that intraoperative UAS increases the durability of reusable fURSs [43], [44]. In both studies, the authors highlighted the UAS’s ability to reduce stress at the tip of the instrument during introduction, while also imposing minimal stress on the scope during repeated withdrawal and insertion; they proposed that these were the key reasons why UAS use increases fURS durability. Conversely, Hosny et al [45] emphasized that there is a risk of damaging the distal deflection tip against the UAS, which increases the risk of fURS damage. To maximize the potential of the UAS to increase fURS durability, both components should be extracted together while keeping the distal end of the fURS out of the UAS under visualization.

Two studies have investigated the use of other types of ureteroscopes in selected situations to preserve the reusable fURS and decrease the risk of damage [46], [47]. Defidio et al [46] used a standardized technique where a 9.5Fr semirigid ureteroscope was introduced first for ureteral optical predilation and examination of the upper urinary tract, followed by the introduction of an fURS. This approach contributed to increased durability of the reusable fURS in their facility. Furthermore, Ventimiglia et al [47] tested the effect of using a single-use fURS for complex procedures—where there is a concern for damaging the fURS—on the durability of a reusable fURS. The authors found that after introducing a single-use fURS for complex procedures, the center was able to increase the number of procedures before reusable fURS repair by 40%. Taguchi et al [35] investigated the association between perioperative factors and the risk of fURS damage using multivariable regression, and found that presence of a UAS, degree loss of upward flexion during a case, and safety wire usage were associated with fURS repair. Attention to these or similar risk factors can help identify the types of cases and circumstances where a single-use fURS may be a cost-effective alternative to increase the durability of reusable equipment.

#### 3.4.1 Limitations

While the data presented in this study are informative for decision-making, several limitations should be considered. The main shortcoming of this review is the differences in the quality and research designs of published evidence. First, the existing literature is inconsistent with regard to the fURS handling protocols that influence the repair rate. For example, some practices might use older fURSs, have dedicated urology-specific cleaning personnel versus general equipment maintenance staff, or be able to invest in physician education on proper handling of an fURS. Moreover, the included studies often did not account for whether an fURS previously required repair and was primarily from academic centers (often associated with more complex cases and training of residents), making it difficult to compare results across different facilities. This might explain why the heterogeneity for the pooled estimates decreased after dividing study sites into regions, since guidelines in care and handling of fURSs may be more comparable within regions (Fig. 2). Second, the included studies typically used consistent definitions of what constituted “major” repairs within each study; however, the subgroup analysis of major repairs should be interpreted carefully as definitions among studies could vary. Third, there is the possibility of observation bias in prospective studies of fURS durability and handling during the study period, which may influence the reported repair rate. Fourth, Egger’s test of small study effects was not significant, but the heterogeneity across studies may suggest uncertainty for the pooled estimates. The statistical heterogeneity of the pooled estimate decreased when dividing the studies into regions and into sheath use in majority versus minority of cases. In particular, the subgroup analyses of studies with different sheath use indicated less uncertainty than the overall pooled estimate. We did not analyze repair rates by manufacturing brand (1) as most studies did not consider manufacturing brand as part of their study design and instead used the brand and model of devices that were available, and (2) because of a small sample size. Hence, this study is unable to comment on manufacturing quality. Last, there are limitations regarding the subgroup analyses carried out in this study. Some were based on small sample sizes, which may reduce the validity. Despite these limitations, our findings regarding the true prevalence of fURS repair frequency are novel, and may help surgeons and practices make evidence-based decisions about purchasing and clinical indications for the use of reusable versus single-use fURSs.

## 4 Conclusions

This systematic review and meta-analysis of fURS repairs revealed an overall repair rate of 6.5%, corresponding to an average of 15 ureteroscopy procedures in between fURS repairs and at a cost of 441 USD per procedure. Subgroup analyses indicated a rate of 4.5% for major repairs and a higher repair rate for digital fURSs. Ureteroscopy practices should consider factors influencing fURS breakage rates and repair costs to optimize the use of reusable versus disposable devices. Additional studies may help identify the circumstances and procedures where single-use fURSs would offer improved cost effectiveness and reusable fURS durability.

  ***Author contributions*:** Dinah K. Rindorf had full access to all the data in the study and takes responsibility for the integrity of the data and the accuracy of the data analysis.

*Study concept and design*: Koo, Rindorf.

*Acquisition of data*: Rindorf, Larsen, Koo, Tailly.

*Analysis and interpretation of data*: Rindorf, Koo.

*Drafting of the manuscript*: Rindorf, Koo.

*Critical revision of the manuscript for important intellectual content*: Koo, Tailly, Kamphuis, Somani, Traxer.

*Statistical analysis*: Rindorf.

*Obtaining funding*: None.

*Administrative, technical, or material support*: Rindorf.

*Supervision*: Koo.

*Other*: None.

  ***Financial disclosures*:** Dinah K. Rindorf certifies that all conflicts of interest, including specific financial interests and relationships and affiliations relevant to the subject matter or materials discussed in the manuscript (eg, employment/affiliation, grants or funding, consultancies, honoraria, stock ownership or options, expert testimony, royalties, or patents filed, received, or pending), are the following: Dinah K. Rindorf is employed by Ambu and Sara Larsen was previously employed by Ambu. Thomas Tailly is a consultant for Boston Scientific, Cook Medical, and Karl Storz, and an advisory board member for Ambu. Guido M. Kamphuis is an advisory board member for Ambu, consultant for Boston Scientific and Olympus, and lecturer in educational courses for Coloplast and EMS. Olivier Traxer is a consultant for Boston Scientific, Coloplast, IPG Medical, Olympus, and Rocamed. Bhaskar K. Somani and Kevin Koo declared no conflicts of interest.

  **Funding*/Support and role of the sponsor*:** The authors did not receive any specific funding for this research from any funding agency in the public, commercial, or not-for-profit sectors.
