# Supplementary material for: Repair Rate and Associated Costs of Reusable Flexible Ureteroscopes: A Systematic Review and Meta-analysis
Source: Eur Urol Open Sci. 2022 Jan 29;37:64–72. doi: 10.1016/j.euros.2021.12.013 (PMC8810356; doi:10.1016/j.euros.2021.12.013)
Supplement: Supplementary Data 1 [file mmc1.docx]

**Supplementary material**

Forest plots of the pooled estimate of fURS repair rate for subgroups. The green diamond represents the overall 95% CI.

**
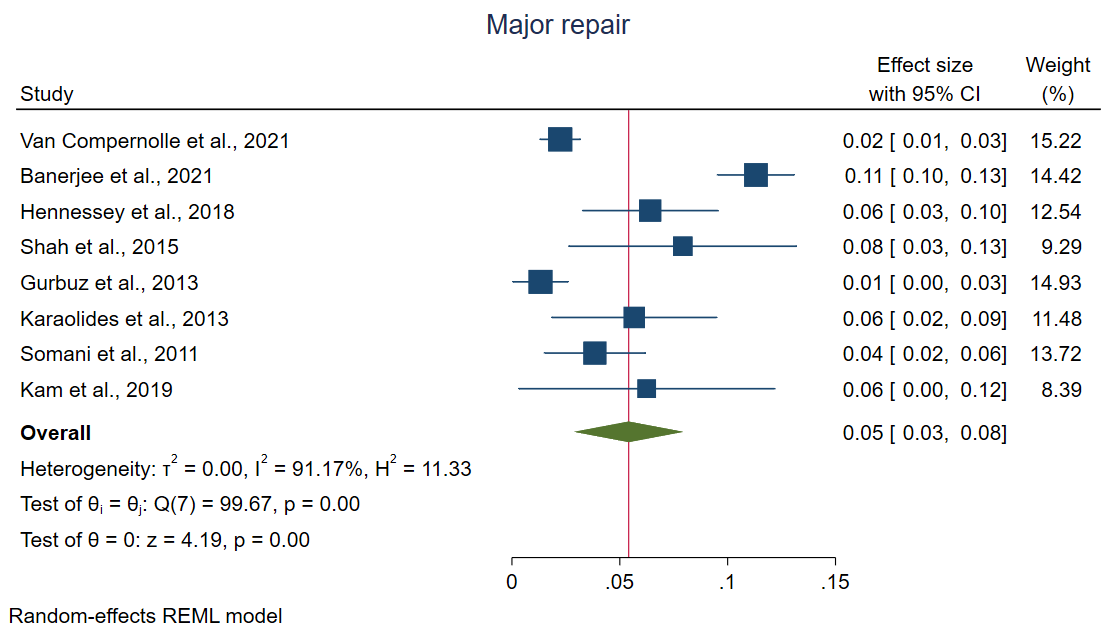
**

**
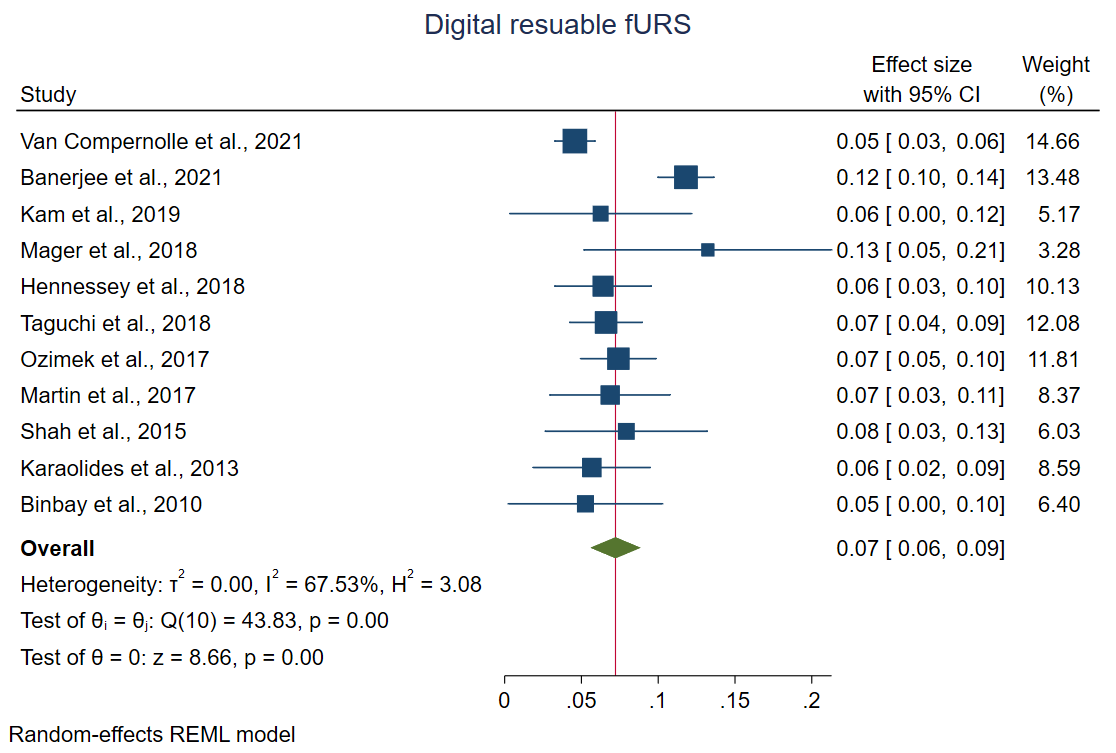
**

**
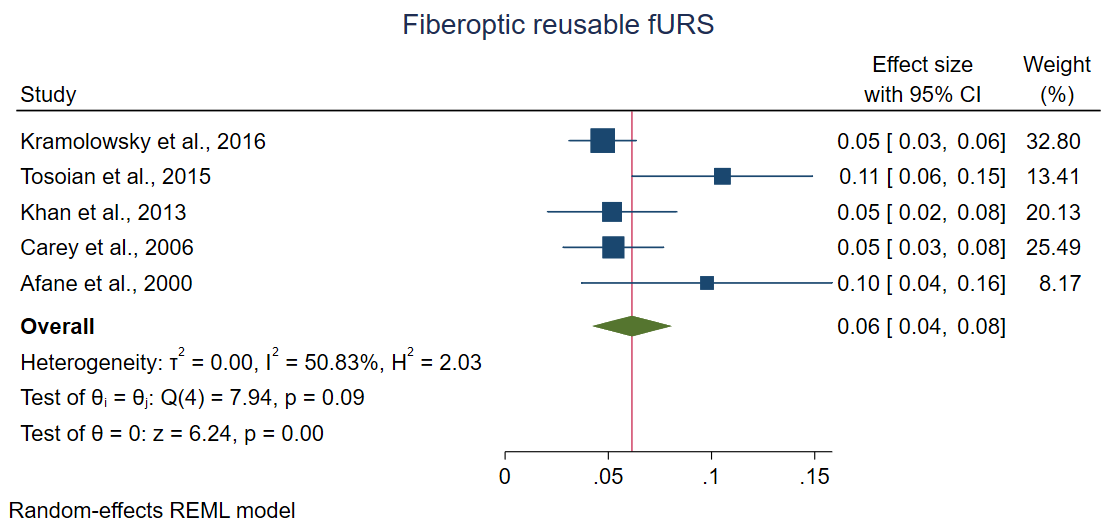
**

**
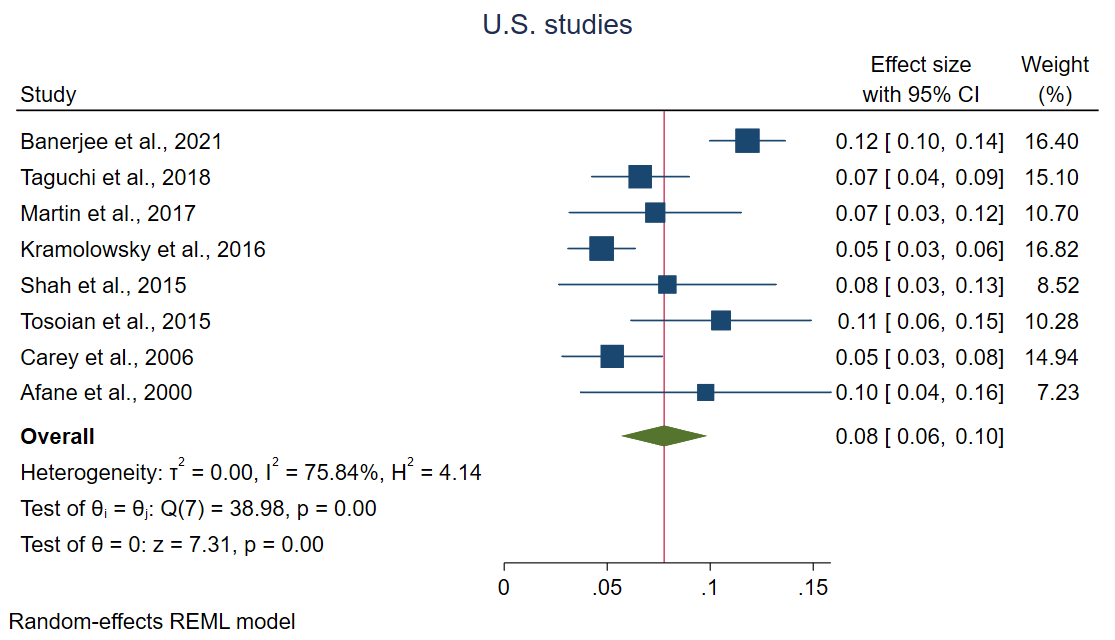
**

**
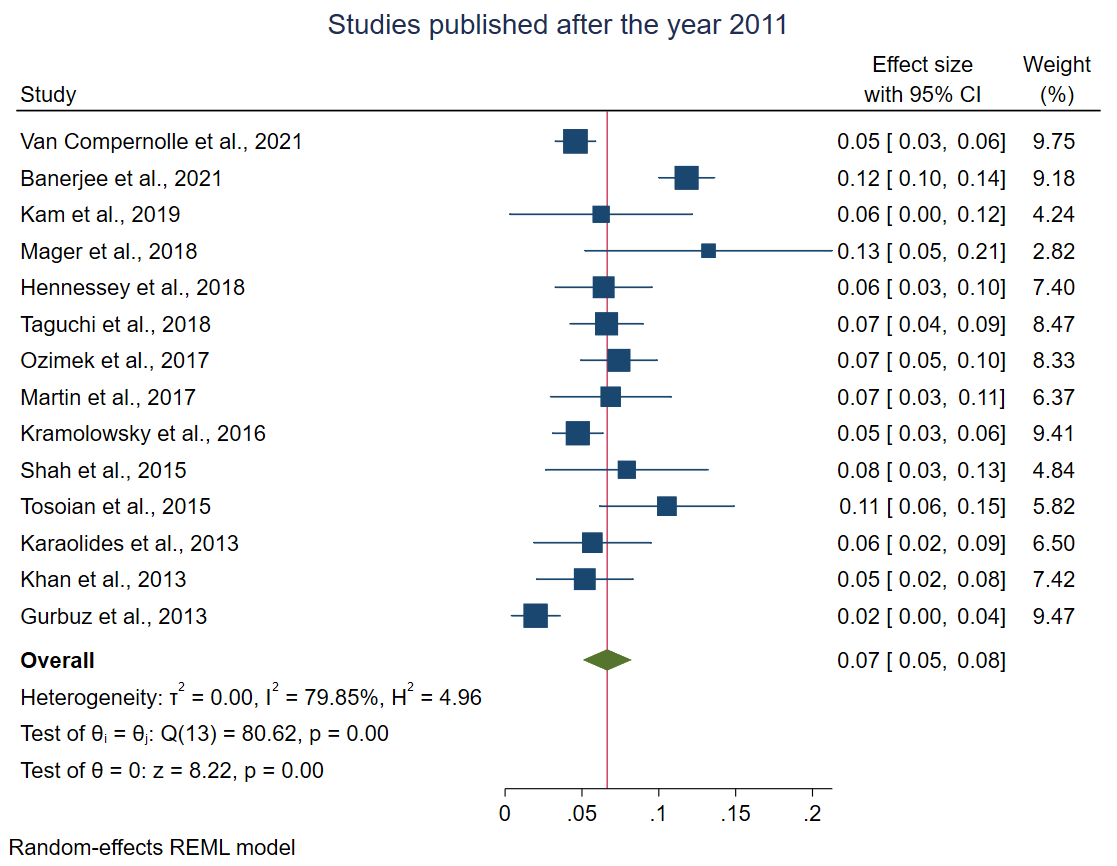
**

**
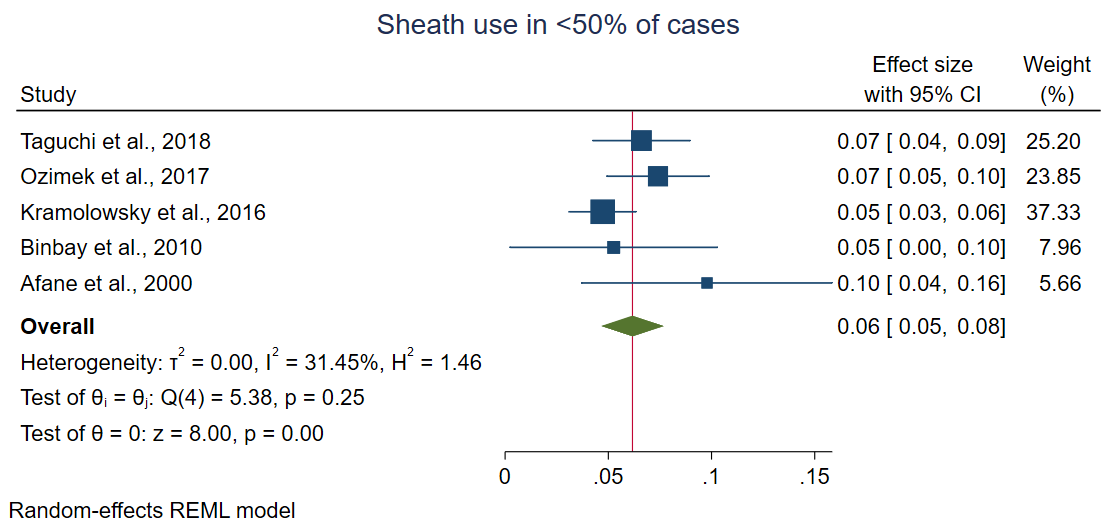
**

**
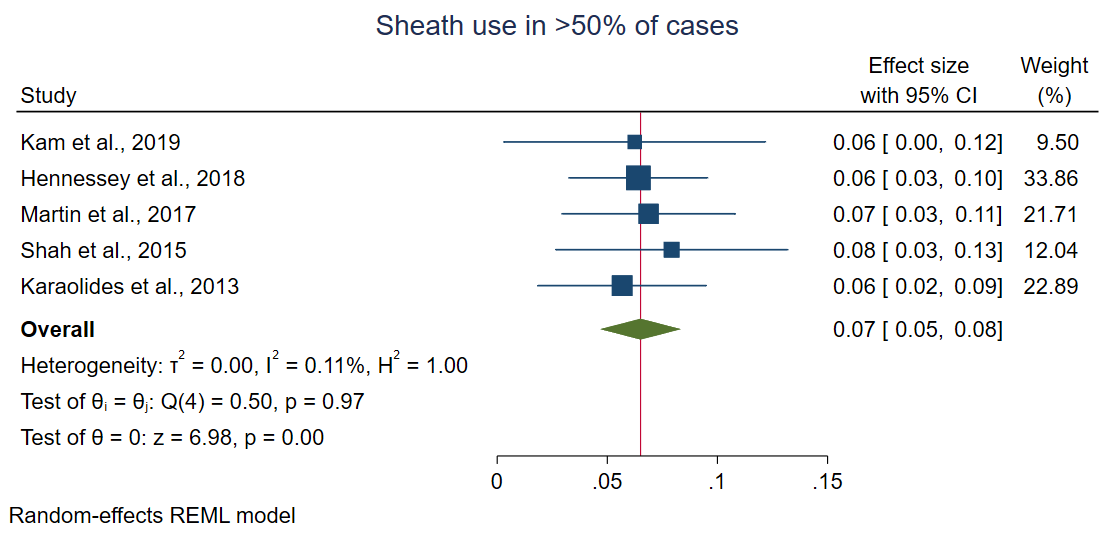
**
